# Case Report: One-stop procedure for atrial fibrillation patients with dextrocardia

**DOI:** 10.3389/fcvm.2024.1454976

**Published:** 2024-11-08

**Authors:** Lin Chungyun, Cui Kaijun

**Affiliations:** Department of Cardiology, West China Hospital, Sichuan University, Chengdu, Sichuan, China

**Keywords:** atrial fibrillation, mirror dextrocardia, radiofrequency ablation (RFA), left atrial appendage (LAA) closure, cerebral infarction—complications

## Abstract

We present the case of an elderly woman with congenital dextrocardia who experienced severe palpitations. An electrocardiogram revealed paroxysmal atrial fibrillation, and her medical history unveiled cerebral infarction and renal failure. In the treatment of paroxysmal atrial fibrillation, anticoagulation is a key requirement. Although non-vitamin K antagonist oral anticoagulants are recommended by guidelines, their efficacy is compromised in patients undergoing dialysis due to renal metabolism. In addition, warfarin may cause fluctuations in international normalized ratio, which is not conducive to controlling symptoms of cerebral infarction. To improve the patient's quality of life, we focused on addressing the unique challenges posed by dextrocardia in a one-stop procedure.

## Introduction

In patients with atrial fibrillation (AF) who are at high risk for both bleeding and stroke, the combination of radiofrequency ablation (RFA) and left atrial appendage closure (LAAC) presents itself as a viable alternative, particularly for those with non-valvular atrial fibrillation (NVAF) (1). Furthermore, existing literature underscores its long-term efficacy in mitigating stroke risk among afflicted individuals (2).

In addition, the presence of mirror dextrocardia, an exceedingly rare congenital cardiac anomaly, further complicates the challenge. While the major arterial structures may remain unaltered, the anomalous horizontal orientation or mirror-image reversal poses formidable obstacles during an intervention.

## Case report

The patient under discussion, a 69-year-old obese woman, found herself in need of renal replacement therapy due to renal failure, a condition accompanied by recurrent episodes of heart palpitations. These palpitations occurred many times during the treatment process. A bedside electrocardiogram (ECG) revealed paroxysmal atrial fibrillation. Notably, her medical history further unveiled a complex interplay of ailments, including congenital mirror dextrocardia with sinus inversus, although she showed no signs of vascular anomalies, hypertension, or diabetes. In addition, her medical history included a diagnosis of medullary thyroid carcinoma (MTC), a condition that exhibited improvement following a thyroidectomy. During strict treatment, she had previously presented at the emergency department with symptoms including limb rigidity, trismus, binocular gaze, and syncope. Over the course of treatment, she gradually regained consciousness. Non-vitamin K antagonist oral anticoagulants (NOACs) are not recommended for patients undergoing long-term hemodialysis. Significant fluctuations in the international normalized ratio (INR) raise concerns about the efficacy and safety of the anticoagulation treatment.

## Procedure

**Figure 1:**
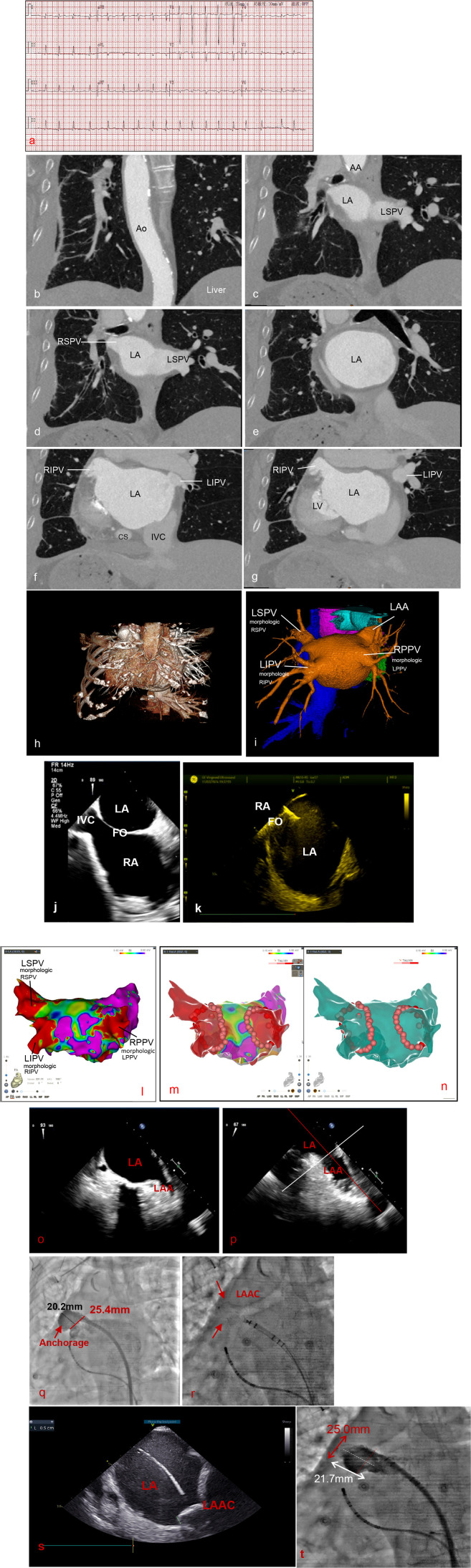
**(a)** Patient's ECG before ablation showing AF with rapid ventricular rate. **(b–g)** Preprocedural computed tomography images showing the sequence from the Ao to LV section in the coronal view. **(h,i)** 3D reconstruction of cardiac CT in AP and PA views. **(j)** Section of the ME bicaval view by TEE before the procedure. **(k)** Short-axis section of the left atrium. The patient had a mirror image transposition of the cardiovascular arteriovenous system. During the procedure, the septal puncture was guided by x-ray imaging. No “jump phenomenon” was seen, making septal localization difficult. Instead, we searched for the FO from the patient's INF view under ICE guidance. The needle was inserted, and the contrast agent was quickly applied, suggesting cloudy visualization. The puncture was successful. **(l)** 3D electroanatomic mapping of the left atrium in the PA view, describing the voltage pattern of the endocardium before ablation (red <0.1 mV, purple ≥0.5 mV). The low-voltage areas of the LA in the anterior and posterior views are scattered, which may indicate fibrosis. **(m,n)** Voltage pattern after ablation, visualizing the ablation path by tag points. The reduction in low-voltage areas around the ring of bilateral pulmonary veins is suggested. **(o,p)** Preoperative transesophageal echocardiography. **(q,r)** Guidance sheath used in the intervention, with intraoperative measurement of left atrial pressure >10 mmHg. Windbag structure in the anteroposterior view **(o,p)**. Measurement of the left atrial appendage opening at 25.4 mm and a depth of 20.2 mm (Supplementary Video 1) **(q)**. WATCHMAN FLX 31 mm closure device is deployed with a compression ratio of about 19% (Supplementary Video 2) **(r)**. **(s)** Short-axis section in intracardiac echocardiography showing shoulder protrusion of about 5 mm. **(t)** Left atrial appendage angiography after LAAC placement (Supplementary Video 3). Ao, aorta; AA, aortic arch; IVC, inferior vena cava; CS, coronary sinus. RPPV, right primary pulmonary vein; LIPV, left inferior pulmonary vein; RIPV, right inferior pulmonary vein; LSPV, left superior pulmonary vein; RSPV, right superior pulmonary vein; AP, anteroposterior; PA, posteroanterior. ME, middle esophageal; INF, inferior.

The patient underwent an ECG and three-dimensional (3D) reconstruction of cardiac computed tomography (CT) before the intervention (as shown in Figures 1a–i). Preoperative routine transesophageal echocardiography (TEE) was performed to observe the morphology of the atrial septum (Figure 1j) and to exclude the presence of intracardiac thrombus (Figures 1o,p). On the day of the interventional procedure, the INR of the patient was maintained within the range of 2.0–3.0. Warfarin administration was discontinued in the morning, and heparin bridging anticoagulation was commenced, ensuring an activated clotting time (ACT) exceeding 300 s.

## Ablation procedure

Following local anesthesia, the left femoral vein was punctured to place the coronary sinus electrode; a transseptal puncture was then performed via the left femoral vein under intracardiac echocardiography (ICE) guidance, with one VISIGO long sheath inserted into the left atrium (Figure 1k). Through a single transseptal access, the Pentary (Johnson & Johnson) matrix mapping electrode was placed; the left atrium and pulmonary vein geometry model was constructed under CARTO 3D mapping system guidance in combination with left atrium and pulmonary vein angiography (Figure 1l). Using the ThermoCool® SmartTouch catheter (Johnson & Johnson), the ostium of the pulmonary vein was identified, and a meticulous point-by-point ablation isolation procedure was executed on both the anterior and posterior aspects of the left and right pulmonary vein ostia, thus achieving bilateral pulmonary vein isolation lines (Figures 1m,n). After a 20-min observation period, no extra-pulmonary vein (PV) triggers of AF were mapped; subsequently, 20 mg of intravenous adenosine triphosphate (ATP) was administered for repeat verification of the bidirectional block.

## LAAC procedure

Once the catheter ablation (CA) was completed, the transseptal sheath was exchanged for a 14F WATCHMAN access sheath. Selective LAA angiography was performed using a 6F pigtail catheter, which was positioned inside the left atrial appendage (LAA). The diameter and morphology of the LAA were measured and evaluated using ICE and selective LAA angiography in the right anterior oblique 30° with caudal 20° view (Figure 1q; Supplementary Video 1). The appropriate LAAC device was selected according to the guidelines. The delivery sheath was advanced along the pigtail catheter until the distal marker reached the tip of the LAA (Figure 1r; Supplementary Video 2). After removing the pigtail catheter, the WATCHMAN FLX™ device was advanced to the predetermined position. The device was subsequently released by gradually withdrawing the delivery sheath. The following criteria had to be met before releasing the device: (A) accurate placement of the device in the LAA was confirmed by imaging (Figure 1s); (B) blood flow into the LAA was absent or the residual flow of the device was <5 mm (as determined by ICE and contrast-enhanced imaging) (Figure 1t); and (C) a tug test confirmed that the device was stable (Supplementary Video 3).

Uninterrupted systemic anticoagulation was recommended for at least 3 months following the ablation, while antiarrhythmic drugs were not administered. One month after the ablation, the patient remained asymptomatic, without dyspnea on exertion, and maintained sinus rhythm. A TEE report shows no thrombus formation around the LAAC device, and the residual blood flow around the device was measured at 3 mm.

## Discussion

Congenital mirror dextrocardia with sinus inversus is a rare congenital condition with an incidence of approximately 0.01% (3). We present a case involving an elderly woman patient with renal failure, paroxysmal atrial fibrillation, and cerebral infarction. Due to the limitations of renal function affecting drug metabolism, NOACs were considered unsuitable for long-term dialysis treatment (4). In addition, managing atrial fibrillation and cerebral infarction with warfarin poses significant challenges, complicating the implementation of a treatment plan.

While many cases have reported successful outcomes with catheter ablation and LAAC (5, 6), there are no documented instances of these procedures being performed in combination in patients with dextrocardia. A standardized catheter ablation procedure typically employs electrocardiogram characteristics and anatomical landmarks identified via fluoroscopy to guide catheter placement. In our case, after puncturing the left femoral vein (as opposed to the right femoral vein for a normally positioned heart), the inverted x-ray imaging angle cannot overcome the challenges posed by anatomical variations of the fossa ovalis (FO). The thick or fibrotic nature of the FO, along with the absence of its mechanical protrusion, makes the puncture more difficult. Moreover, the structural complexity of the LAA makes it difficult to navigate using fluoroscopy alone. TEE usually requires general anesthesia and orotracheal intubation, which can expose patients to undetected acute stroke, pneumonia, or severe esophageal lesions. Instead, recent studies from a large, nationwide database have shown that the use of ICE offers significant procedural effectiveness (ICE vs. non-ICE: 98.3% vs. 97.9%) while also reducing complications (ICE vs. non-ICE: 1.7% vs. 4.6%) during ablation procedures (7, 8).

Compared to x-ray guidance, ICE offers significant advantages for interatrial septal puncture and LAA occlusion procedures (9, 10). In our case, postoperative TEE revealed no shunting at the interatrial septum. In addition, TEE also indicated a 3-mm leak from the occluder, which is within with the accepted threshold (less than 5.5 mm) (10).

Dextrocardia is a rare condition often associated with vascular access or anatomical anomalies (11, 12). Varying degrees of structural abnormalities may increase the severity of arrhythmias in these patients. Therefore, judicious management of imaging tools and the development of interventional strategies are particularly important. ICE-guided puncture allows the operator to perform real-time maneuvers at the desired angles, enhancing both the safety and effectiveness of the procedure. This technique is especially suitable for the interventional treatment of complex cardiac structural abnormalities, aligning with conclusions established in other studies (13, 14).

## Conclusion

In the past, interventions such as catheter ablation and LAAC in patients with mirror dextrocardia relied on high-quality imaging and positioning during the procedure. ICE allows for real-time visualization of the location of FO and LAA occlusion, dynamically displaying the spatial relationship between the catheter and key anatomical structures, making the one-stop procedure effective and secure. However, more evidence is needed to achieve widespread adoption of this approach in the future.

## Data Availability

The original contributions presented in the study are included in the article/Supplementary Material, further inquiries can be directed to the corresponding author.
